# Biogeographical variation in diurnal behaviour of *Acanthaster planci* versus *Acanthaster* cf. *solaris*

**DOI:** 10.1371/journal.pone.0228796

**Published:** 2020-02-20

**Authors:** Deborah Burn, Samuel Matthews, Ciemon F. Caballes, Josie F. Chandler, Morgan S. Pratchett

**Affiliations:** 1 ARC Centre of Excellence for Coral Reef Studies, James Cook University, Townsville, Queensland, Australia; 2 Ultra Coral Australia, Paget, Queensland, Australia; 3 Gili Lankanfushi Resort, Lankanfushi Island, North Male Atoll, Maldives; Australian Bureau of Agricultural and Resource Economics and Sciences, AUSTRALIA

## Abstract

Crown-of-thorns starfish (CoTS; *Acanthaster* spp.) are among the most extensively studied coral reef taxa, largely owing to their devastating impacts on live coral cover during population outbreaks. Much of this research has however, been conducted in the western Pacific, although it is now apparent that there are several distinct species of *Acanthaster* spp. across the Indo-Pacific. The purpose of this study was to test for biogeographical variation in behaviour, comparing between *Acanthaster planci* at Lankanfushi Island in the Maldives and *Acanthaster* cf. *solaris* at Rib Reef on Australia’s Great Barrier Reef. The extent to which CoTS were exposed (cf. concealed within or beneath coral substrates) was substantially higher (63.14%) for *A*. *planci* at Lankanfushi Island, compared to 28.55% for *A*. cf. *solaris* at Rib Reef, regardless of time of day. More importantly, only 52% of individuals were exposed at night at Rib Reef compared to >97% at reefs around Lankanfushi Island. Biogeographic variation in the behaviour of *Acanthaster* spp. was independent of differences in the size structure of starfish and coral cover at specific study sites, but may be attributable to other environmental factors such as habitat complexity or prey availability. This is the first study to explicitly test for biogeographical differences in the biology and behaviour of *Acanthaster* spp., potentially linked to species-specific differences in the causes and explanations of population outbreaks. However, we did not find evidence at this stage of differences in behavior among regions, rather behavioural differences observed were most likely products of different environments.

## Introduction

Crown-of-thorns starfishes (CoTS; *Acanthaster* spp.) have gained considerable notoriety over the last few decades following outbreaks throughout the Indo-Pacific [1–8]. Along with anthropogenic climate change, outbreaks of CoTS are a major contributor to coral loss and reef degradation [9], causing extensive coral mortality [10] and shifts in the biological and physical structure of coral reef habitats [11–14]. Unsurprisingly, CoTS are among the most extensively studied organisms from coral reef environments [2], though the majority of this research has been conducted in the western Pacific, and particularly on Australia’s Great Barrier Reef (GBR) [15]. Extensive research into causes and consequences of recurring outbreaks on the GBR (e.g., [16–19]) has been foundational to understanding and managing outbreaks globally. However, outbreaks on the GBR are unlike outbreaks in many other locations [20–21] and the generality of conclusions drawn, and broader relevance of learning from GBR studies may be limited.

CoTS were initially described from several different geographical regions across the Indo-Pacific and assigned distinct species names [22]. However, all coral reef species were subsequently synonymised as *A*. *planci*, as distinct from *Acanthaster brevispinus* that occurs in deep-water non-reefal environments [23]. Extensive molecular sampling has since revealed marked genetic differences [24] consistent with at least 4 distinct species [25]. Most importantly, *Acanthaster planci* [26], which is restricted to the northern Indian Ocean, is readily distinguishable from the Pacific species, nominally, *Acanthaster solaris* [22] in both phylogenetics [24] and appearance [15]. Species-specific differences in biology and behaviour may account for geographic variation in the occurrence of outbreaks, and their impacts on reef ecosystems [27].

Ecological effects of CoTS on coral assemblages are influenced by their individual behaviour, including feeding preferences and diurnal activity patterns [28, 29]. In general, CoTS preferentially consume *Acropora* and *Montipora* corals (reviewed by [27]), leading to localised depletion of these corals, which often dominate coral assemblages in the Indo west-Pacific. Reported departures from this typical feeding pattern (e.g. [30, 31]) are attributed to localised differences in the size and abundance of CoTS, as well as regional variation in the structure of coral assemblages [28]. Similarly, there are reported biogeographical differences in diurnal activity patterns [28]. For example, in the Red Sea, Ormond and Campbell [32] reported that CoTS are almost exclusively nocturnal, whereas CoTS in the Pacific are often active during the day [33], especially during population outbreaks. Variation in diurnal activity patterns is often attributed to the individual size and density of CoTS, whereby CoTS become less cryptic as they increase in size [3, 23, 28] and increasingly feed both day and night when food becomes limiting [33]. Geographical variation in behaviour may however, reflect inter-specific differences in the biology and behaviour of distinct CoTS species, which was potentially overlooked when all CoTS were considered to be the same species.

The purpose of this study was to explicitly test for biogeographical differences in behaviour of CoTS potentially linked to inter-specific differences, comparing between *A*. *planci* from the northern Indian Ocean and *A*. cf. *solaris* from the western Pacific. In particular, we documented the number of starfish that were out in the open (as opposed to hiding on the underside of corals or deep within the reef matrix) and whether they were observed feeding, resting or moving, at different times of day. This study is important because it represents the first explicit test of inter-specific differences in the biology and behaviour of CoTS, potentially accounting for biogeographical differences in the incidence and severity of CoTS outbreaks [34], while also focussing on *A*. *planci* from the northern Indian Ocean, which is particularly underrepresented in previous research on CoTS [35]. The extent to which CoTS are cryptic versus exposed has ramifications for their detectability, which constrains understanding and management of population outbreaks [36], but may also influence their ecological impacts [30].

## Materials & methods

### *Acanthaster planci* in the Maldives

**Fig 1:**
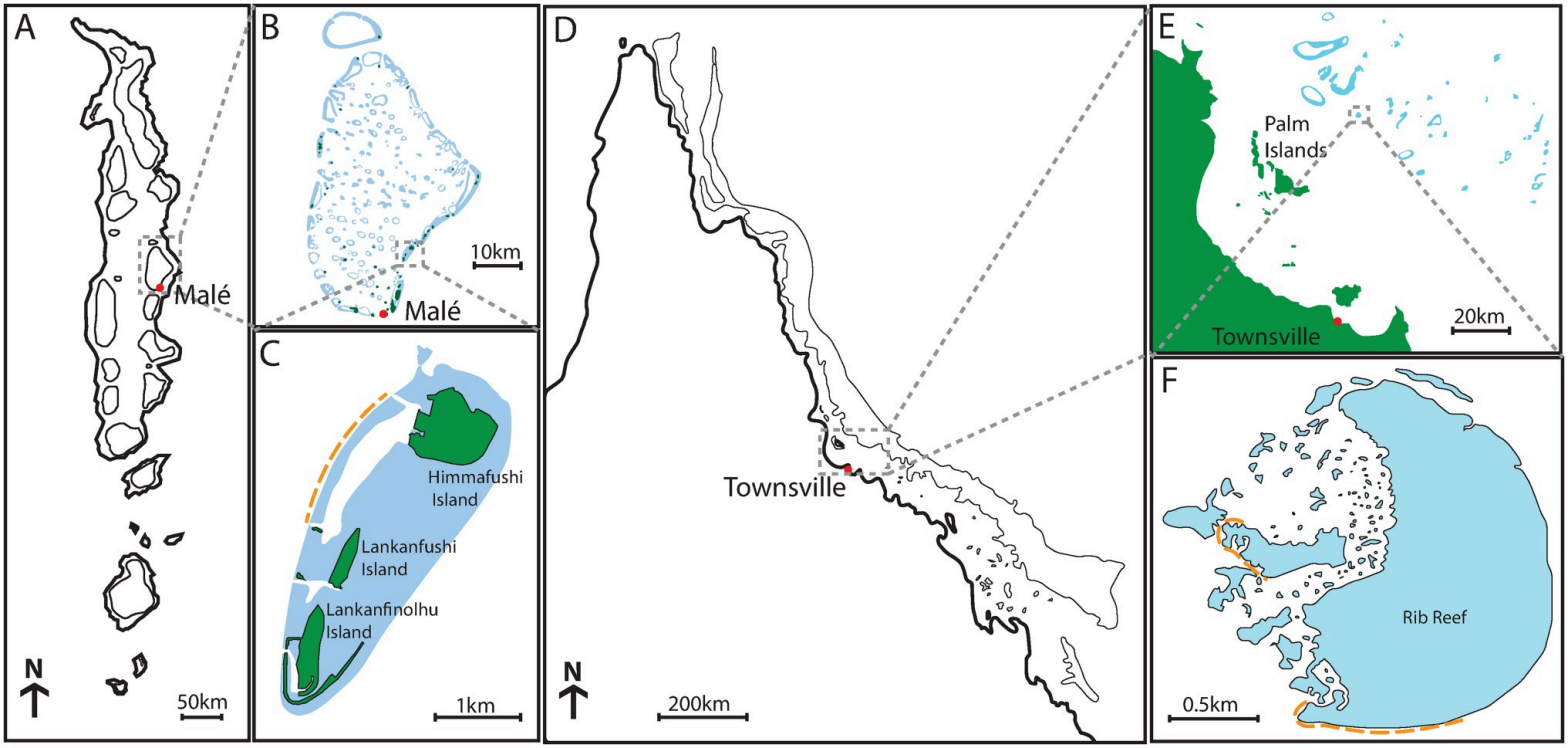
Study site locations highlighted in orange at both Lankanfushi Island and at Rib Reef. A) The Republic of Maldives located in the Indian Ocean. B) North Malé Atoll. C) Study site located at Lankanfushi Island in the Himmafushi-Lankanfushi-Lankanfinolhu Island Group. D) The Great Barrier Reef located off Australia’s Queensland coast. E) Central Great Barrier Reef. F) Study site located at Rib Reef.

Behaviour of *A*. *planci* was studied in the Maldives, in the central Indian Ocean. The earliest reports of outbreaks of *A*. *planci* on Maldivian reefs are from the 1970s [37], though the first well-documented outbreak started in 1987 in North Male Atoll, spreading to Gaafu Alifu/Gaafu Dhaalu Atoll in the south [38]. Elevated densities of *A*. *planci* were again recorded in the Maldives in 1999 to 2007 [39]. Most recently, severe and widespread outbreaks of *A*.*planci* have affected South Male and Ari Atolls [7, 35], starting in 2014. This study was undertaken in March 2017, on the western facing fringing reef of the Lankanfinolhu-Lankanfushi-Himmafushi island group located on the eastern outer rim reef of North Male Atoll in central Maldives (Fig 1). The site was chosen due to the high density of *A*.*planci* in comparison with nearby reefs. Despite localised culling between October 2015 and June 2016, outbreaks of *A*. *planci* had caused extensive coral loss in the study area, which was further compounded by severe coral bleaching in May 2016 [35, 40, 41].

To test for diurnal changes in the behaviour of CoTS at Lankanfushi Island, three permanent replicate 100m transects were established on both the reef crest (8m) and the reef slope (15m) on the inner atoll rim reef between Lankanfinolhu Island and Himmafushi Island (4°17’53.0”N 73°33’13.7”E). During surveys, which were conducted randomly at various times of day and night from February 25^th^ to March 29^th^, 2017, two divers carefully searched for CoTS within a 1m wide belt on either side of the transect line, along each of the six transects. Divers swam parallel to each other, whilst visually checking crevices and overhangs across the 2m belt to allow for different angles of visual perception, paying particular attention to areas where feeding scars were observed to ensure the likelihood of detection of both exposed and cryptic animals. In all, each of the six transects were surveyed at four different times of day. Times were grouped into categories including morning (surveys between 06:30–10:00), midday (11:30–13:00), afternoon (14:00–17:15) and night (18:00–22:00). Surveys were undertaken with permission from Gili Lankanfushi Resort, who manage the section of reef surveyed, negating the need for a permit.

### *Acanthaster* cf. *solaris* on the Great Barrier Reef

Comparable research on *A*. cf. *solaris* was conducted at Rib Reef, in the central GBR, Australia (Fig 1) (18°29’11.1”S 146°52’33.8”E). The first documented outbreak of CoTS on the GBR occurred in 1962, with subsequent outbreaks starting in 1979, 1993 and 2009–10 [27]. In all instances, outbreaks have started in the northern GBR, between Cooktown and Cairns, before spreading north and south [21, 42, 43] affecting mid-shelf reefs along the entire length of the GBR. This study was conducted from December 2016 to May 2017, when outbreaks were concentrated in the area between Cairns and Townsville [34]. High densities of *A*. cf. *solaris* caused extensive coral depletion at Rib Reef during 2015 and 2016, though patches of high coral cover remained, mostly on distinct patch reefs on the leeward, north-west margin, as well as inside the lagoon.

Reef access was much more limited at Rib Reef, compared to Lankanfushi Island, in the Maldives. As such, two-four permanent replicate 50m transects were established during each of three distinct reef visits, with sampling conducted in a different location on each visit to account for prevailing conditions and changes in the distribution and abundance of CoTS. In all, five transects were surveyed on reef crest habitats (3m) and five on slope habitats (6m). CoTS were surveyed within 2.5m either side of transects on the reef crest, where there was high cover of tabular *Acropora* (live and dead). In deeper, less complex habitats, surveys were extended to 5m either side of the transect line to ensure enough CoTS were captured to allow for statistical analysis. As densities of CoTS were analysed, rather than abundance per site, this did not confound results. Each of the ten transects were surveyed at several different times of the day, including at least one survey during daylight hours and one survey after dark. Survey times were placed into the same time categories as the Maldives surveys for comparative analysis. Surveys at Rib Reef were undertaken under Marine Parks permit G15/37363.1 issued by the Great Barrier Reef Marine Park Authority.

### Quantifying diurnal patterns of behaviour

All CoTS recorded within the area of belt transects at both Lankanfushi Island, Maldives and Rib Reef, central GBR, were measured (diameter from arm tip to opposite arm tip) *in situ* to the nearest cm. The extent to which individuals were visible from above (or at 90° perpendicular to the substrate on particularly steep sections of reef) was then recorded to the nearest 10%. There were however, two distinct modes in these data, where the majority (85%) of starfish were either completely (100%) exposed or entirely concealed (0% exposed) beneath the reef substrate and only visible from an acute angle. As such, we categorised starfish as either cryptic, where ≤50% of the starfish was exposed, versus exposed where >50% of the starfish was visible from above. In addition to exposure, we also recorded whether each starfish was moving versus stationary, based on sustained movement of the body of the starfish. For starfish that were not moving, one or more arms were lifted away until it was possible to see whether the stomach was everted. Starfish with stomachs partially or fully everted were recorded as feeding, whereas individuals with stomachs completely retracted were considered to be resting.

### Coral cover

Coral cover was determined along each of the transects, which were demarcated by a fibreglass tape, at both locations before CoTS surveys began. Benthic cover was determined to the lowest possible taxonomic level, with scleractinian corals identified to genus and growth form, using the point intercept method every 50cm.

### Statistical analysis

To assess the variation in CoTS density between reef locations and habitats, a linear model with gaussian error distribution was constructed, to which the time of observation (Time: Morning, Midday, Afternoon, Night) and interaction terms for location:Habitat and location:Time was also included. Similarly, a linear model was constructed to determine variations in the size of CoTS individuals between reef locations, habitats and time of day, using the same interaction terms as above. Analysis of variance and post-hoc comparisons (using R package ‘lsmeans’) of factor levels was also conducted to determine the significance of these variations in density and size. Potential interactions were tested and retained if they improved model fit on the basis of the corrected Akaike information criterion [44]. To test for differences between the proportions of CoTS observed exhibiting different behaviours between reefs, Chi-Squared two sample tests for proportions were conducted. Additionally, to assess the differences between the coral communities at the two reef locations, non-metric multidimensional scaling and an analysis of similarity (ANOSIM) were conducted using R 3.5.1 [45] using the ‘vegan’ (v2.5.6, [46]) package, using a Bray-Curtis dissimilarity matrix and Wisconsin double standardization.

To test for variation in the cryptic behaviour of CoTS, we constructed a Bayesian generalised linear model to investigate the effects of 7 different predictor variables; location (Lankanfushi Island versus Rib Reef), habitat (crest and slope), body size, coral cover, behaviour (moving or stationary), time of day (morning, midday, afternoon, night) and CoTS density (per 100m^2^). Exposure was recorded as a binary variable (either cryptic or exposed) and thus modelled using a Bernoulli distribution using the *R* package ‘brms’ [47]. All potential interactions were tested and retained if they improved model fit on the basis of the widely applicable information criterion [48]. Effects plots with 95% credible intervals were produced to show the magnitude and direction of the effect for each variable reported as log odds ratios. Marginal effects plots were also produced to show how CoTS exposure varied with respect to each of the predictor variables individually.

## Results

**Fig 2:**
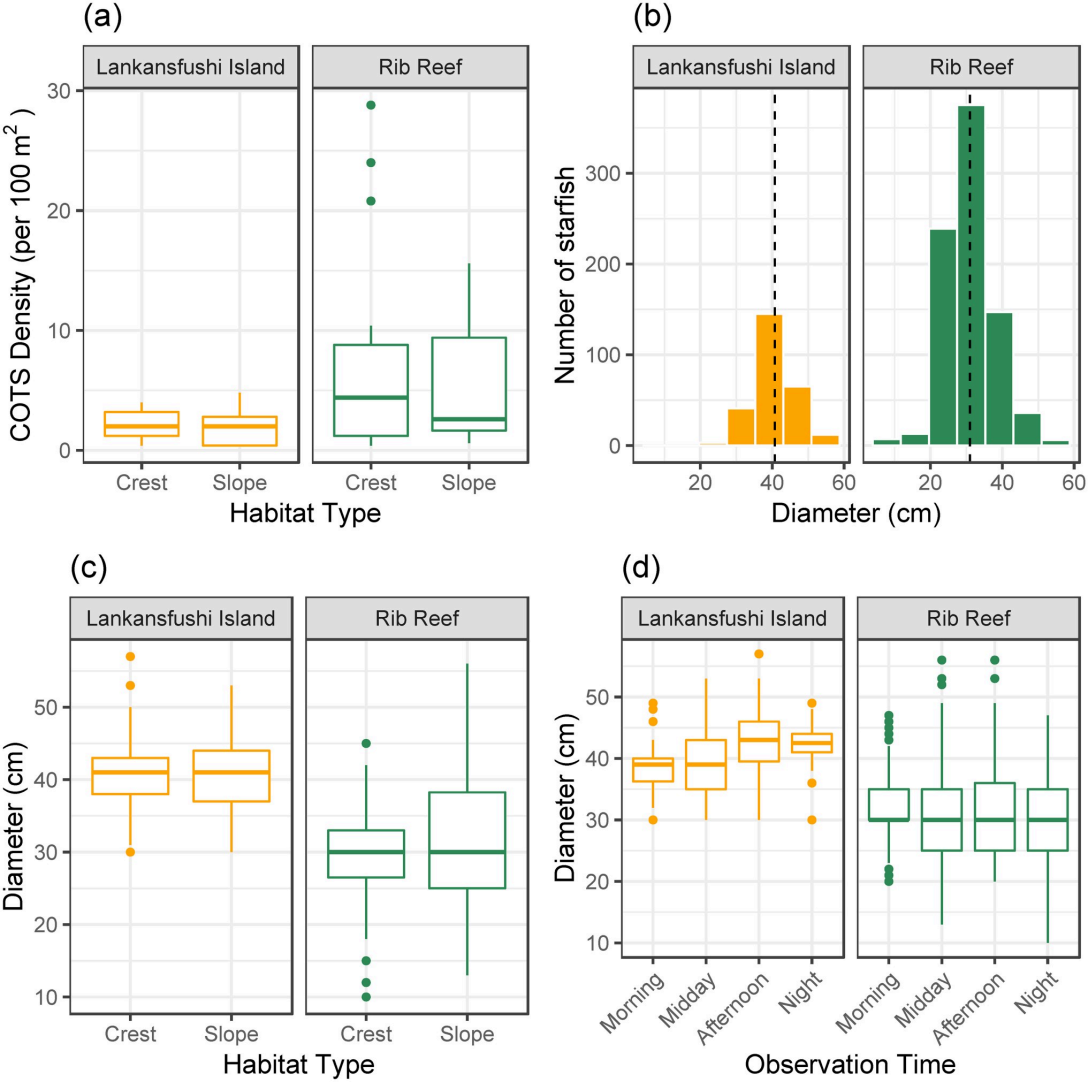
Comparisons between Lankanfushi Island and Rib Reef. A) Boxplot of CoTS per 100m^2^ between habitat types. B) Frequency histogram of diameter of observed individuals. The mean is represented by the dotted line. C) Boxplot of diameter of observed individuals between habitats. D) Boxplot of diameter of observed individuals among observation time categories.

Average densities of CoTS recorded at Rib Reef (6.29 starfish per 100m^2^ ±1.08 SE) were significantly higher than recorded at Lankanfushi Island, Maldives (2.65 starfish per 100m^2^ ±0.31 SE; F_1,63_ = 8.29, p<0.01) however, there was no difference detected among habitats (F_1,63_ = 0.81, p = 0.37) or time of day (F_3,63_ = 0.92, p = 0.44) (Fig 2). The size of *A*. cf. *solaris* ranged between 10-56cm and 22-57cm at Rib Reef and Lankanfushi Island, respectively (Fig 2b). The average size of CoTS was significantly smaller at Rib Reef (31.01cm total diameter ±0.25 SE) compared to Lankanfushi Island (40.73cm total diameter ±0.43 SE), owing to the increased number of small starfish at Rib Reef (F_1,967_ = 269.3, p<0.0001), however there were also significant interactions between reef and observation time (Fig 2d: F_3,967_ = 7.23, p<0.0001) and reef and habitat (Fig 2c: F_1,967_ = 6.65, p<0.0001)). At Rib Reef, slightly larger starfish were found on the slope (32.12cm ± 0.39SE) compared to the crest (29.86cm ± 0.30SE: t_1,967_ = -5.45, p<0.0001) and starfish observed in the morning were significantly larger compared to all other time periods (Morning-Midday: t_1,967_ = 3.19, p = 0.008; Morning-Afternoon: t_1,967_ = 3.75, p = 0.001; Morning-Night: t_1,967_, p = 0.0002) however, these differences were very low in magnitude (1-2cm, Fig 2). At Lankanfushi Island, there was no significant difference between habitats (t_1,967_ = 0.21, p = 0.83). In contrast to Rib Reef, slightly smaller starfish were observed in the morning compared to the night and afternoon (Morning-Afternoon: t_1,967_ = -2.85, p = 0.023; Morning-Afternoon: t_1,967_ = -2.41, p = 0.076), however these differences were also low in magnitude (~3cm) (Fig 2).

**Fig 3:**
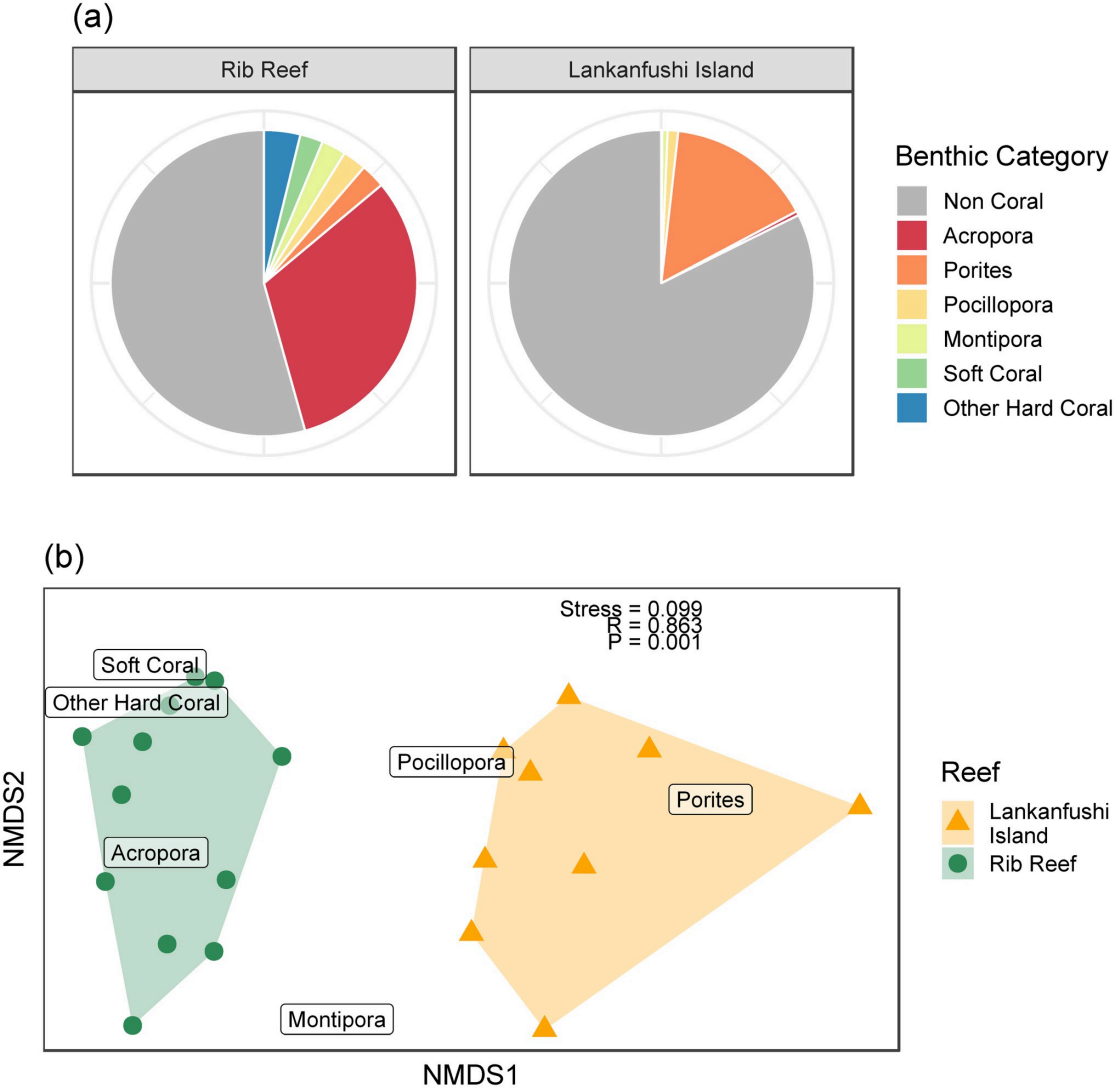
Coral cover and composition. A) Mean percent cover of the benthic community at Lankanfushi Island and Rib Reef. B) Non-metric multidimensional scaling and ANOSIM results for benthic community composition comparisons between the two study sites.

The coral composition was significantly different between the two reefs (ANOSIM: R = 0.863, p = 0.001), with *Porites* being the most abundant coral genus at Lankanfushi Island (15.44% ± 6.38SE), while coral cover at Rib Reef was dominated by the preferred prey *Acropora* (31.75% ± 4.88SE) (Fig 3a and 3b).

Overall, CoTS were exposed (where >50% of the animal was visible from directly above) in only 37.14% (410/1104) of records. The proportion of CoTS exposed was however, substantially higher at Lankanfushi Island (63.14%) compared to Rib Reef (28.55%), regardless of time of day. The proportion of exposed CoTS increased through the day at both locations, with lowest levels recorded in the morning (33.97% at Lankanfushi Island and 13.78% at Rib Reef) and highest levels of exposure recorded at night (97.47% at Lankanfushi Island and 52.09% at Rib Reef).

**Fig 4:**
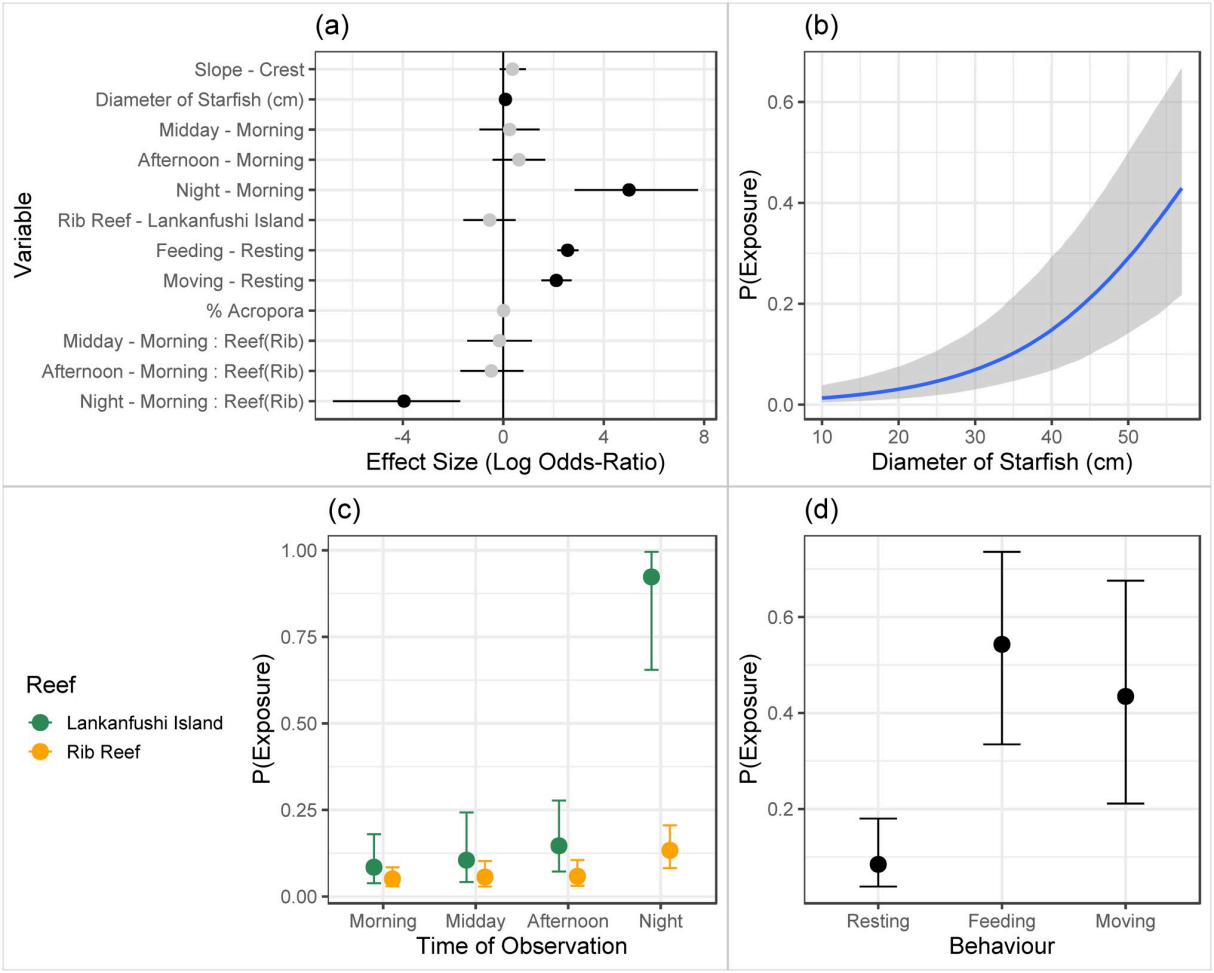
A) Effects plot showing the mean posterior effect size and 95% credible intervals (as log odds) of each predictor variable in the full model (Eqn 1). Black dots indicate variables with "significant" effects. B) Partial effect of diameter on the probability of exposure (P(exposure)). C) Partial effects of this interaction between observation time and reef location on P(exposure). D) Partial effect of behaviour on P(exposure).

The best model (Bayesian generalised linear model) to account for variation in cryptic behaviour of CoTS across both locations included an interaction between reef (Lankanfushi Island versus Rib Reef) and time of day (morning, midday, afternoon, night), percent *Acropora* at each site, as well as the size (total diameter), and the behaviour of starfish (whether starfish were resting, feeding or moving) (Fig 4). At both locations, the probability that CoTS were exposed rather than cryptic was significantly greater at night as opposed to during the day (2.58[1.46,3.97], p<0.05); log odds posterior mean and 95% highest posterior density intervals calculated by contrasting night observations to the next most exposed time; afternoon (P value is inferred as the HPD intervals do not overlap 0). While exposure was not significantly higher at Lankanfushi Island across all time periods (0.54[1.59, 0.49], p>0.05), there was a significant interaction between reef location and time of day with the differences between night and afternoon being much more pronounced at Lankanfushi Island (4.19 [2.04, 7.42], p<0.05) compared to Rib Reef (0.96 [0.39, 1.52]). Furthermore, post hoc analyses revealed that the differences in exposure between Rib Reef and Lankanfushi Island were only significant during night time observations (Fig 4c). The probability of exposure increased with increasing size of CoTS (0.086[0.059, 0.11], p<0.05), in a manner that was consistent across locations (Fig 4b). Unsurprisingly, individuals were also more likely to be exposed when feeding (2.56[2.15, 2.99], p<0.05) or moving (2.11[1.52, 2.72], p<0.05) compared to resting (Fig 4d). The probability of exposure did not vary with respect to habitat (Slope-Crest = 0.37[-0.14, 0.90], p = 0.16) or percent *Acropora* (0.012[-0.002, 0.03], p = 0.09) (Fig 4a). However, when Reef was excluded from the model, percent *Acropora* performed the same function as Reef location, with an overall significant negative effect of percent *Acropora* (-0.02 [-0.035, -0.004], p<0.05) however, this model had poorer performance overall according to widely applicable information criterion.

The proportion of *A*. *planci* that were observed feeding (as opposed to moving or resting) was higher at Lankanfushi Island (44.03%) compared to *A*. cf. *solaris* at Rib Reef (35.00%: χ^2^ = 4.31, df = 1, p = 0.019). Conversely, a significantly higher proportion of CoTS were recorded resting at Rib Reef (56.99%) compared to Lankanfushi Island (46.54%; χ^2^ = 5.48, df = 1, p = 0.0097), whereas the proportion of starfish actively moving was similar between locations (9.43% at Lankanfushi Island and 8.02% at Rib Reef;; χ^2^ = 0.19, df = 1, p = 0.67). It was apparent that the proportion of individuals observed feeding at Lankanfushi Island was more consistent throughout the day (ranging from 38–64%) and was significantly higher during the midday surveys (64.5%) compared to night time (38.2%; χ^2^ = 3.49, df = 1, p = 0.031), whereas the incidence of feeding by *A*. cf. *solaris* at Rib Reef increased throughout the day. The proportion of *A*. cf. *solaris* recorded feeding at night (65.4%) was more than twice that recorded at any time during the day (13.4–26.7%; χ^2^ = 58.27, df = 1, p<0.0001, compared to next highest proportion, afternoon). To test whether this result was attributable to the large number of smaller (<25cm total diameter) starfish at Rib Reef, whereby smaller starfish may be more cryptic and tend to feed nocturnally [23, 3, 28], we compared behaviour of starfish ≥25 cm total diameter, but this did not alter the overall pattern; even for among *A*. cf. *solaris* ≥25 cm total diameter, the proportion of individuals feeding increased from 13.02% in the morning to 62.92% at night.

## Discussion

Given their important contribution to degradation and disturbances on coral reefs, there has been extensive and increasing research into the biology and ecology of *Acanthaster* spp. over the last three decades (reviewed by [15, 27]), with a particular focus on reproductive biology, outbreak dynamics and population control. However, most of this research has been conducted in the western Pacific, and mainly on the GBR (e.g., [49]). Despite marked genetic differences between the Pacific and Indian Ocean populations of CoTS, which were first revealed 20 years ago [50], there has been comparatively limited research on the biology or behaviour of CoTS from the Indian Ocean (but see [15, 35]). It is implicitly assumed that all putative species (at least four different species; [25]) from different bioregions behave in much the same way. Whilst this study has revealed potentially important differences in the diurnal behaviour between *A*. *planci* from Lankanfushi Island in the Maldives and *A*. cf. *solaris* from Rib Reef on the GBR, this is likely attributable to varying environmental conditions, rather than species-specific traits.

The most pronounced difference observed between *A*. *planci* from the Maldives and *A*. cf. *solaris* on the GBR were higher levels of cryptic behaviour for CoTS on the GBR, especially at night. The proportion of starfish exposed at night was higher than during the day at both locations, but for *A*. cf. *solaris* on the GBR only 52% of individuals were found out in the open during the night, while a similar proportion remained largely concealed within and/ or beneath live or dead corals. In contrast, at Lankanfushi Island in the Maldives, virtually all individuals of *A*. *planci* (>97%) were fully exposed at night. Similarly, Ormond and Campbell [32] reported marked increases in activity and exposure at night for *Acanthaster* spp. from the Red Sea, where CoTS were reported to feed almost exclusively at night. Increased nocturnal exposure in the Maldives did not translate into higher feeding incidence, though movement was greatest at night. At Rib Reef on the GBR however, greater exposure at night was accompanied by pronounced nocturnal feeding. In some locations, *Acanthaster* spp. are reported to switch from feeding mainly at night to feeding day and night with the onset of population outbreaks (e.g., [51]). These shifts in diurnal behaviour may occur in response to increased food competition or food limitation, though we’d expect to see increased diurnal feeding at higher CoTS densities. In our study, diurnal feeding was more apparent at Lankanfushi Island, despite lower CoTS densities compared to Rib Reef. Coral cover was however, much lower at Lankanfushi Island (21.4%) than at Rib Reef (43.4%), and a pronounced difference in preferred prey existed between the two locations as live coral cover was dominated by *Acropora* at Rib Reef and *Porites* at Lankanfushi Island. To fully explain behavioural changes that may occur due to changes in CoTS densities and depletion of prey sources, we would need to observe the behaviour of *A*. *planci* versus *A*. cf. *solaris* through the entire course of population outbreaks at multiple reefs.

The differences in behaviour of CoTS between the two locations, particularly the increased levels of exposure at Lankanfushi Island, is most likely linked to the lower levels of complex branching corals (e.g., branching and tabular *Acropora*) at this location. Reef habitats with high cover of complex corals are likely to offer more opportunities for *Acanthaster* spp. to remain concealed, even while continuing to feed. While coral composition and habitat (slope vs crest) had no effect on the probability of exposure for CoTS that were recorded, the effect of percent *Acropora* was likely masked in our model by the inclusion of reef location. Indeed, our results showed percent *Acropora* performing the same function as reef location, when the latter was removed from the model. Despite this, it is also likely that detectability itself varies with coral cover and habitat complexity [36]. It was very apparent, for example, that a greater number of CoTS were detected during nocturnal surveys on the shallow reef crest compared to diurnal surveys at the same location. While it is possible that these starfish are moving among habitats, it is also possible that the starfish are so cryptic during the day that they completely avoid detection. Study sites at Lankanfushi Island were surveyed in the aftermath of the 2016 bleaching event [40], which combined with sustained high densities of *A*. *planci* since 2015, have caused extensive depletion of acroporid and pocilloporid corals, which are generally preferred prey of *Acanthaster* spp. [27]. The lack of nutritional value afforded by the remaining non-preferred coral species (predominantly massive *Porites*) [52], combined with declines in structural complexity following the inevitable degradation of branching coral skeletons, may explain the generally higher levels of exposure and consistently high levels of feeding behaviour of CoTS at Lankanfushi Island (cf. Rib Reef) regardless of time of day. Conversely, the higher abundance of preferred prey species (mostly *Acropora*) at Rib Reef likely explains higher densities and wider size ranges of CoTS recorded at this location. Despite being subject to moderate bleaching in 2016 and 2017 [53] and sustained outbreaks of *A*. cf. *solaris*, there was still moderate cover of *Acropora* spp. at Rib Reef, allowing prey to not only sustain large densities of adult starfish, but also facilitate secondary waves of recruitment and replenishment.

The most likely explanation for the diurnal variation in exposure, and specifically increases in cryptic behaviour of CoTS during daylight hours, is that predation risk is substantially greater during the day compared to night [33]. The role of predator evasion in explaining probability of exposure is further reinforced by the relationship between body size and probability of exposure across both species and study locations. Therefore, as well as clear differences in topography, regional differences in predation risk may also account for differences in behaviour between locations, rather than it being a reflection of inherent differences between species of *Acanthaster*. Predation risk will vary with size, abundance and composition of potential predators which will, in turn, vary due to inherent biogeographical patterns, as well as overarching influences of anthropogenic extraction and reef degradation [54]. In the Philippines, for example, the proportion of *A*. cf. *solaris* with sublethal injuries, presumed to be caused by sublethal predation, was higher inside a marine reserve compared to nearby areas that were open to fishing [54]. If *Acanthaster* spp. respond to increased predation risk inside marine reserves by being more cryptic, this might lead to differences in detectability, which will need to be considered when comparing density estimates inside versus outside of marine reserves (e.g., [19]). There remains a definite need to quantify predation rates on CoTS at different times of day and in different habitats [15, 55].

This study has revealed differences in behaviour between *A*. *planci* at Lankanfushi Island in the Maldives and *A*. cf. *solaris* at Rib Reef on Australia’s GBR. These preliminary results from just two locations suggest these differences are a product of variation in extrinsic factors, in particular coral prey availability and structural complexity afforded by differences in coral composition, along with possible differences in predation risk. It is important, however, given recognition of species boundaries for *Acanthaster* spp. across the Indo-Pacific [24, 25], that we expect species-specific differences in their biology and behaviour which may, in turn, mean that there are different causes and explanations for population outbreaks in different biogeographic regions. This study is the first to present behavioural data from a population of *A*.*planci* in the Maldives, which is useful for the management and control of CoTS throughout this region, but more work is required to establish whether any behavioural differences are reflective of intrinsic biological differences between species of *Acanthaster*.
